# Arthroscopic bone graft procedure combined with arthroscopic subscapularis augmentation (ASA) for recurrent anterior instability with glenoid bone defect: a cadaver study

**DOI:** 10.1186/s40634-018-0121-0

**Published:** 2018-02-27

**Authors:** Raffaele Russo, Marco Maiotti, Ettore Taverna

**Affiliations:** 1 Orthopedics and Traumatology Unit, Pellegrini Hospital, Via Portamedina alla Pignasecca 41, 80134 Naples, Italy; 2 Sports Medicine Unit & Orthopedic Center, San Giovanni Addolorata Hospital, Via dell’Amba Aradam 9, 00184 Rome, Italy; 3 Orthopedics and Traumatology Unit, Galeazzi Hospital, Via Riccardo Galeazzi 4, 20161, Milan, Italy

**Keywords:** Recurrent shoulder instability, Cadaver study, Arthroscopic bone block procedure, ASA procedure, ASA-BB, Glenoid bone loss, Contact sports

## Abstract

**Background:**

Glenoid bone loss and capsular deficiency represent critical points of arthroscopic Bankart repair failures. The purpose of this study was to evaluate an all-arthroscopic bone block procedure associated with arthroscopic subscapularis augmentation (ASA) for treating gleno-humeral instability with glenoid bone loss (GBL) and anterior capsulo-labral deficiency. Our hypothesis was that these two procedures could be combined arthroscopically. The feasibility of this technique and its reproducibility, and potential neurovascular complications were evaluated.

**Methods:**

A tricortical bone graft was harvested from the cadaveric clavicle, and in one case a Xenograft was used. An anterior-inferior GBL of about 25% was created. Two glenoid tunnels were set up from the posterior to the anterior side using a dedicated bone block guide, and four buttons were used to fix the graft to the glenoid. The subscapularis tenodesis was performed using a suture tape anchor. Afterwards, the shoulder was dissected to study the relationship between all portals and nerves. The size of the bone block, its position on the glenoid and the relationship with the subscapularis tendon were investigated.

**Results:**

In all seven specimens (five left and two right shoulders), the bone block was flush with the cartilage and fixed to the anterior-inferior part of the glenoid. No lesions of the surrounding neurovascular structures were observed. No interference was found between the two bone block tunnels and the anchor tunnel used for the tenodesis.

**Conclusions:**

This study demonstrated the feasibility and reproducibility of this combined arthroscopic technique (bone block associated with ASA) in the treatment of anterior shoulder instability associated with anterior bone loss and anterior capsular deficiency.

## Background

The etiology and patho-mechanics of recurrent gleno-humeral dislocations are not completely known (Alkaduhimi et al., 2016; Arciero et al., 2015; Burkhart & De Beer, 2000; Di Giacomo et al., 2016; Shin et al., 2016; Symeonides, 1989; Symeonides, 1972) and optimal surgical management of anterior shoulder instability remains controversial. Few studies have been carried out on post-traumatic capsular elongation and hyperlaxity or on the healing process of the soft tissue on the glenoid edges after the first dislocation and after capsulolabral repair (Marco et al., 2017; Bonazza et al., 2017). On the contrary, considerable attention has been focused on the correlations among glenoid bone loss, humeral head defects and instability, particularly in the recurrent forms if correlated with engaging Hill-Sachs lesions (Cautiero et al., 2017). Recent studies show the biomechanical aspects of restoring the glenoid width using bone augmentation and the role of the anterior capsule in recentering the humeral head on the glenoid fossa (Arciero et al., 2015; Alvi et al., 2016; Fortun et al., 2016). Furthermore, severe bone lesions of the glenoid rim and the Hill-Sachs defect on the humeral head are associated with poor quality capsular tissues (Arciero et al., 2015; Burkhart & De Beer, 2000; Symeonides, 1972; Cole & Warner, 2000). Different techniques for surgical treatment of traumatic and atraumatic recurrent shoulder instability have been previously described, but arthroscopic Bankart repair is the most popular. The failure rate of this technique is reported to be from 15% to 64%, especially in unselected patients with severe glenoid bone loss (Burkhart & De Beer, 2000; Shin et al., 2016; Degen et al., 2016). The association of glenoid bone loss and capsular inconsistence represents the real limit of a standard arthroscopic anterior capsulorrhaphy or an isolated bone graft procedure (Cole et al., 2000; Field et al., 1999; Kleiner et al., 2016; Lafosse et al., 2007; Provencher et al., 2007). The remplissage technique has been proposed as a support for capsular insufficiency and for engaging Hill Sachs lesions, but the results are controversial, and failures are reported to be from 4% to 15% (Wolf & Arianjam, 2014). The use of a tendon sling made around the subscapularis tendon was proposed to prevent anterior instability in the shoulder, using a hamstring graft and enhancing the anterior rim of the glenoid with the same graft (Klungsøyr et al., 2015). Currently, the open or arthroscopic Latarjet procedures are considered to be the most effective techniques for treatment in cases of severe bone defects and poor-quality anterior soft tissue due to the coracoid transfer and conjoint tendon action, with a recurrency between 0 and 5% (Degen et al., 2016; Lafosse et al., 2007; Wolf & Arianjam, 2014; An et al., 2016; Cassagnaud et al., 2003; Latarjet, 1954; Matton et al., 1992; Russo et al., 1990; Russo et al., 1998; Steffen & Hertel, 2013; Taverna et al., 2006; Torg et al., 1987; Vander Maren et al., 1993). Also the conjoined tendon transfer allows stability restoration with no significant range-of-motion loss and a low recurrence rate (Douoguih et al., 2018).

Open and arthroscopic J-bone graft, considered to be much more anatomical approaches (Pauzenberger et al., 2017), can provide glenoid bone restoration, but certain technical aspects of the graft preparation and glenoid implant have led to a low popularity of those procedures. A new operation, consisting of an all arthroscopic bone graft glenoid augmentation using posterior instruments for glenoid drilling and graft stabilization was described (Taverna et al., 2006; Taverna et al., 2008; Taverna et al., 2014), and it seemed to be more reproducible compared to the J graft technique. The present technique is indicated in presence of a bone defect in patients practicing contact sports. Moreover compared to the Latarjet technique it does not modify the coracoacromial arch, the use of a posterior guide is safer and buttons compression fixation of the graft is more axial.

Considering the recent positive experience using partial subscapularis tenodesis on the glenoid rim, known as arthroscopic subscapularis augmentation (Maiotti & Massoni, 2013; Maiotti et al., 2017; Maiotti et al., 2016; Schröter et al., 2016) (ASA), to treat recurrent anterior instability with capsular inconsistence and moderate glenoid bone loss (GBL), we decided to apply both techniques - ASA and Bone Block (ASA-BB) - in very complex cases in which a glenoid bone defect equal to or greater than 25% is associated with capsular insufficiency. ASA procedure solve the problem of an hyperlax capsule or insufficient tissues, instead of treating it with a simple anterior capsulorraphy. The aim of this cadaver study was to demonstrate the feasibility and reproducibility of the combination of these two techniques in the treatment of severe glenoid bone loss (GBL) associated with anterior capsular insufficiency. Our hypothesis was that these two procedures could be combined arthroscopically.

## Methods

### Specimen preparation

ASA-BB techniques were performed on two right and five left cadaveric shoulders. The mean age was 47.7 ± 8.7 years; no specimen had previous shoulder pathology.

The specimens were fresh-frozen and stored at a temperature of − 20 °C until experiments. The specimens were thawed at room temperature for 24 h prior to the procedure. All procedures were performed in the lateral decubitus position, with the arm at 45° of abduction. The posterior portal for the scope was created 1.0 cm lateral to the standard portal, so 1 cm medial and 5 mm inferior to the acromial edge. This portal was used to insert the posterior guide in a correct position on the glenoid, thus avoiding to do an accessory posterior portal.

Antero-superior and antero-inferior portals were created in the rotator interval, and two 8 mm cannulas were used. The gleno-humeral joint was inspected to assess the integrality of anatomic structures according to Detrisac and Johnson principles (Detrisac & Johnson, 1986). The centering of the humeral head was then checked from the antero-superior portal during full range of external rotation. The anterior capsule from the superior gleno-humeral ligament to the inferior ligament was carefully detached. We created a subtotal lesion of about 2 cm, without any possibility of reattachment to the glenoid rim. Glenoid bone loss of about 25% was created in the sub equatorial area of the glenoid using a motorized burr, and this specific percentage of defect was measured using the distance from the pathological glenoid rim and the bare area as a reference point, assuming that the bare spot of the glenoid is located at the geometric center of the inferior glenoid (Burkhart et al., 2002). After bone defect creation, the humeral head was completely dislocated.

### Bone block procedure

**Fig. 1:**
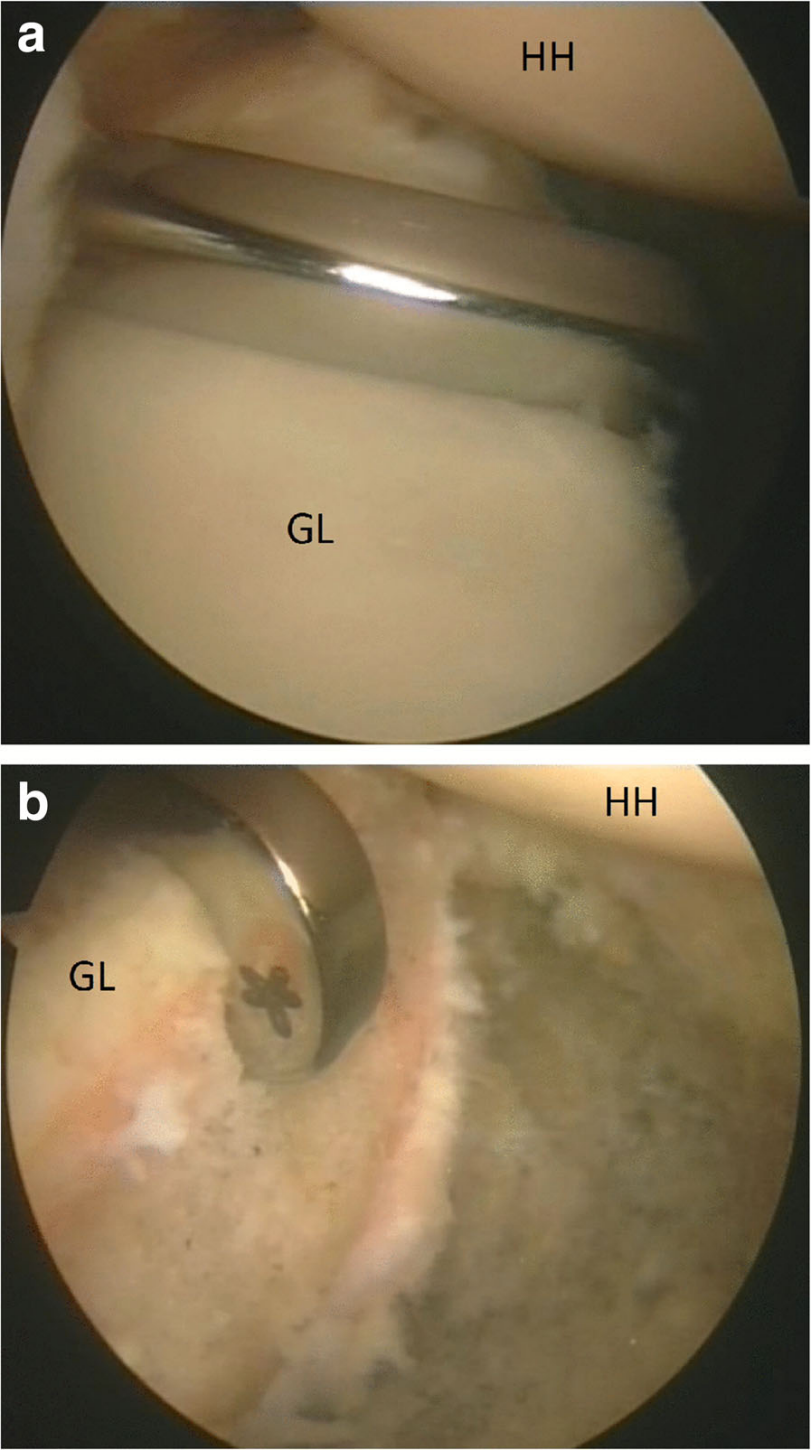
Left shoulder, anterior on the right and posterior on the left, scope from antero-superior portal, hook guide from posterior portal. The Hook guide is parallel to the glenoid surface (**a**) and in the center of bone defect (**b**) (HH: humeral head, GL: glenoid)

**Fig. 2:**
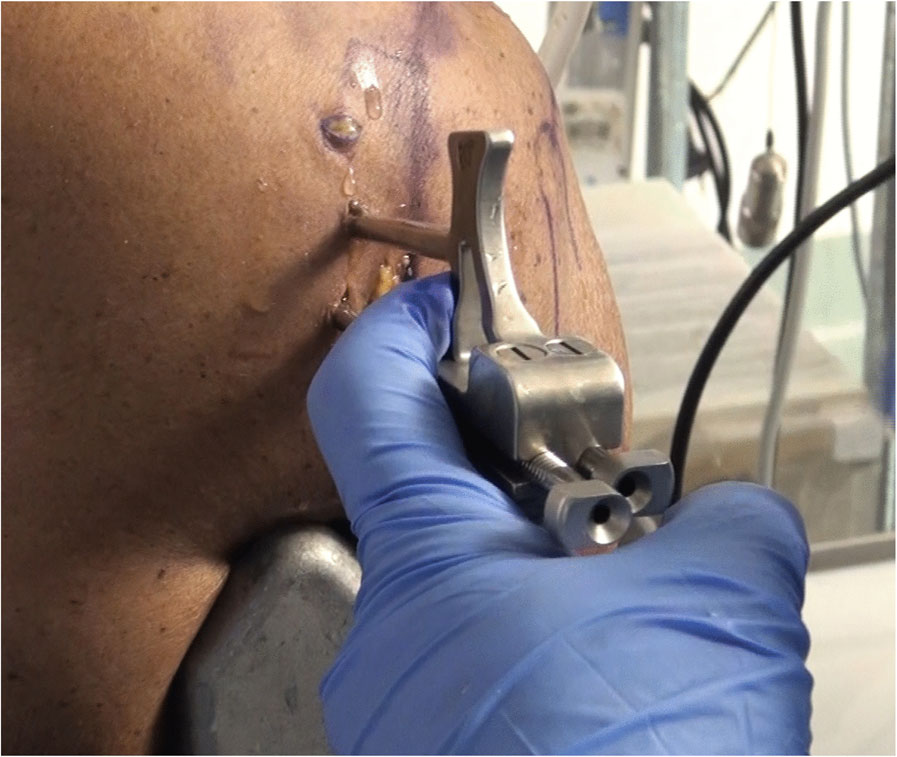
Left shoulder. The guide is positioned posteriorly on the glenoid neck and two bullets are placed in the guide’s holes

The posterior guide was inserted from the posterior portal, using the arthroscope from the anterosuperior portal. Care was taken to introduce the guide parallel to the glenoid surface and to have the bone tunnels perfectly perpendicular to the anterior glenoid neck according to Taverna et al. technique (Taverna et al., 2014). The hook was passed parallel to the glenoid face to avoid damage to the articular surface, and it was advanced over the anterior edge (Fig. 1a). The guide’s hook was placed at the center of the anterior glenoid defect (Fig. 1b). It was mandatory to align the glenoid guide with the posterior and anterior glenoid rims. Once the guide was positioned, a bullet was placed in each hole of the guide (Fig. 2). A 2.8 mm sleeved drill was placed in each bullet and advanced until it came out from the anterior aspect of the glenoid. The drills were placed 5 mm below the cortical edge of the glenoid rim, parallel to one another and 10 mm apart. The inner drill was removed leaving the cannulated outer sleeve. Once drilling was completed, the bullets were removed posteriorly. Flexible looped guidewires were introduced into the joint by passing one wire through each sleeve in a posterior to anterior direction. Each guidewire was retrieved using a loop grasper, which was passed through a cannula introduced through the rotator interval. The wires were separated and stored. The drill sleeves were removed after this step was completed. At this point, the inferior 8 mm anterior cannula was removed and was replaced by a metal cannula with a diameter of 15 mm suitable for passing the graft attached to the two buttons.

**Fig. 3:**
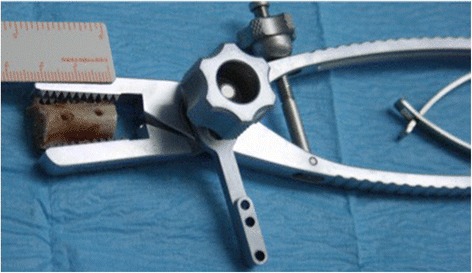
The tricortical clavicular graft

**Fig. 4:**
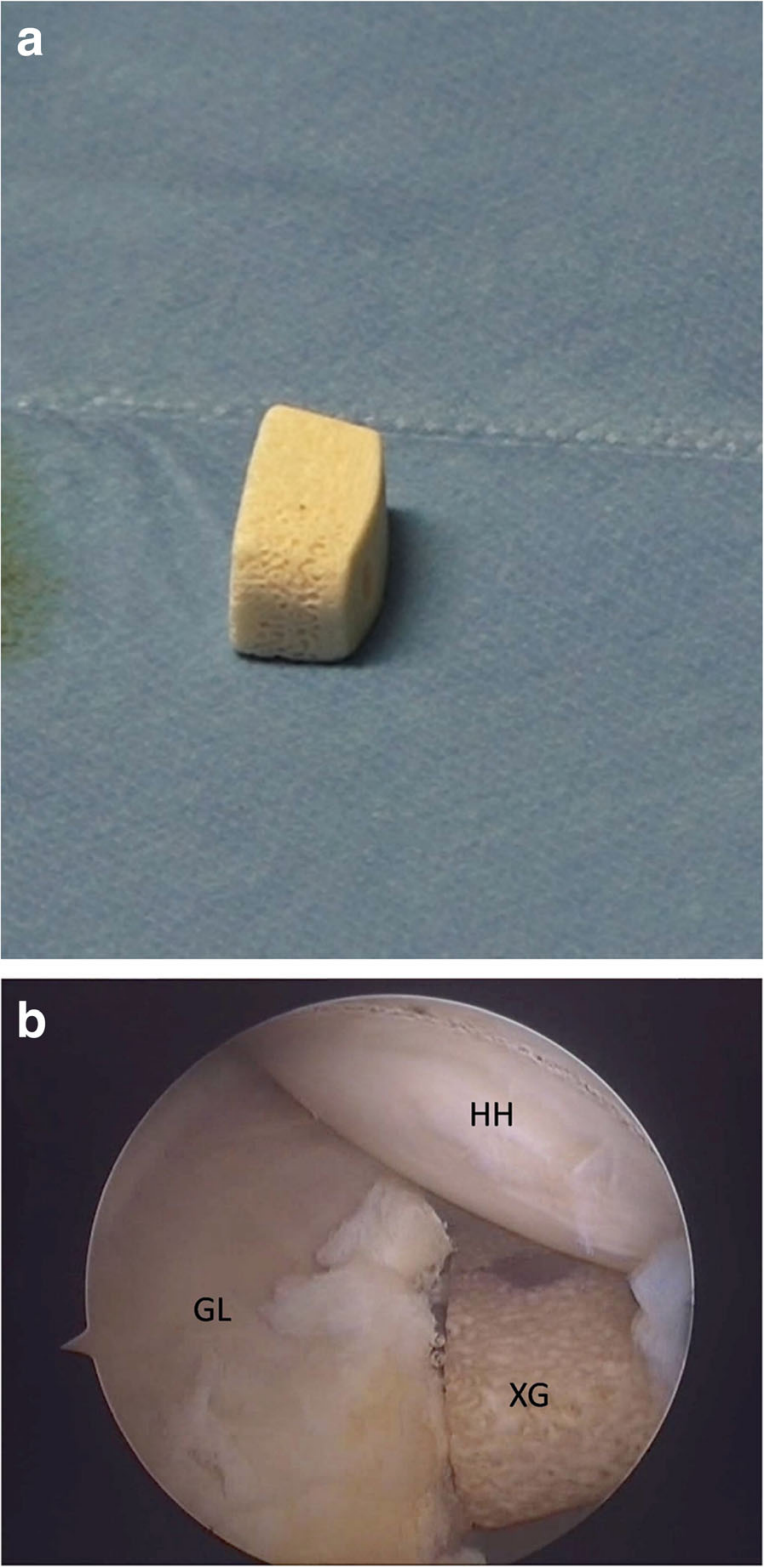
Left shoulder, anterior on the right and posterior on the left, scope from antero-superior portal. The preformed Xenograft (**a**) positioned on the glenoid anterior neck (**b**). (HH: humeral head, GL: glenoid, XG: Xenograft)

In six cases, the tricortical bone graft was harvested from the clavicle because we had only the shoulder specimen. The graft was tailored, cutting off one side of it, so that the one side of the curved cortical aspect was flattened to make it cancellous and compatible with the glenoid bone defect. The graft dimensions were 20 mm × 9 mm × 9 mm. Two 2.8 mm drill holes were made 10 mm apart and 5 mm from each edge of the graft (Fig. 3). The size of the graft is mandatory because harvesting the graft with two metallic buttons increases the thickness such that a larger graft could not slide into a dedicated 15 mm cannula, and the exact position of the two holes was also important**.** The drill was placed through the superior cortex and exited the flattened cancellous side. The holes created corresponded to the distance of the cannulated drill sleeves previously placed in the glenoid neck. In one case, a substitute for bone graft was used. This xenograft (Osteoplant Bioteck SPA) was harvested from the proximal humeral epiphysis of the horse. The graft was completely deantigenated enzymatically, using Zimoteck. The graft can be pre-formed with the holes for the buttons, thus allowing greater precision and reduced operating times (Fig. 4).

**Fig. 5:**
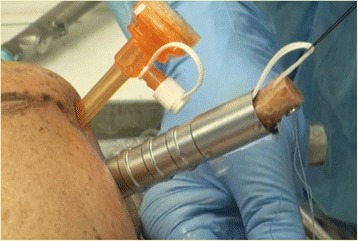
Left shoulder, view from anterior, metal cannula in antero-inferior portal, arthroscopic cannula in antero-superior portal. The graft passed through the metal cannula

Each looped guidewire was fed through the holes prepared in the graft, exiting on the cortical side, after ensuring that the looped guidewires were not tangled within the joint. The anterior implants were fed with the preassembled suture through the end of the looped guidewire using a classic slip-knot. This was achieved by passing the loop of the lead suture through the looped guidewire and feeding the implant through the lead suture. The graft was slid toward the end of the guidewires to lodge the implant. It was important to first retrieve the inferior suture from the antero-inferior portal, and when the whole graft was inside the joint, to retrieve the superior suture. It was important to carefully visualize the position of the graft from the posterior and superior portals. It was possible to correct the graft position by alternatively retrieving the inferior and superior wires using the superior cannula with a probe hook. Anterior round endobuttons (Smith & Nephew, London, England) were advanced until they laid flat on the bone block. The graft was tipped to allow insertion into the 15 mm cannula (Fig. 5) and advanced by pulling the guidewire out posteriorly. The suture advanced the implant until the graft was flush with the glenoid anterior neck, with each suture exiting the skin posteriorly. The graft should not be too medial nor too much lateral, so it must not overflow the articular surface (Allain et al., 1998). The guide hook is placed in the middle of the defect to be sure that the graft will be centered on the defect. The posterior implants were placed on the transporter by advancing the instrument through each eyelet of the posterior round endobutton. We then passed the sutures through the transporter and retracted the transporter to allow the suture to pass through the eyelets of the posterior round endobutton.

The posterior round endobuttons were advanced using a sliding knot until they were flush with the posterior face of the glenoid. A suture tensioner device was used to secure the posterior round endobuttons. Once the implant was tensioned, we secured it with half hitches and cut suture tails (Taverna et al., 2014). After bone block procedure, graft stability was tested and humeral head stability and position were evaluated.

### ASA technique

**Fig. 6:**
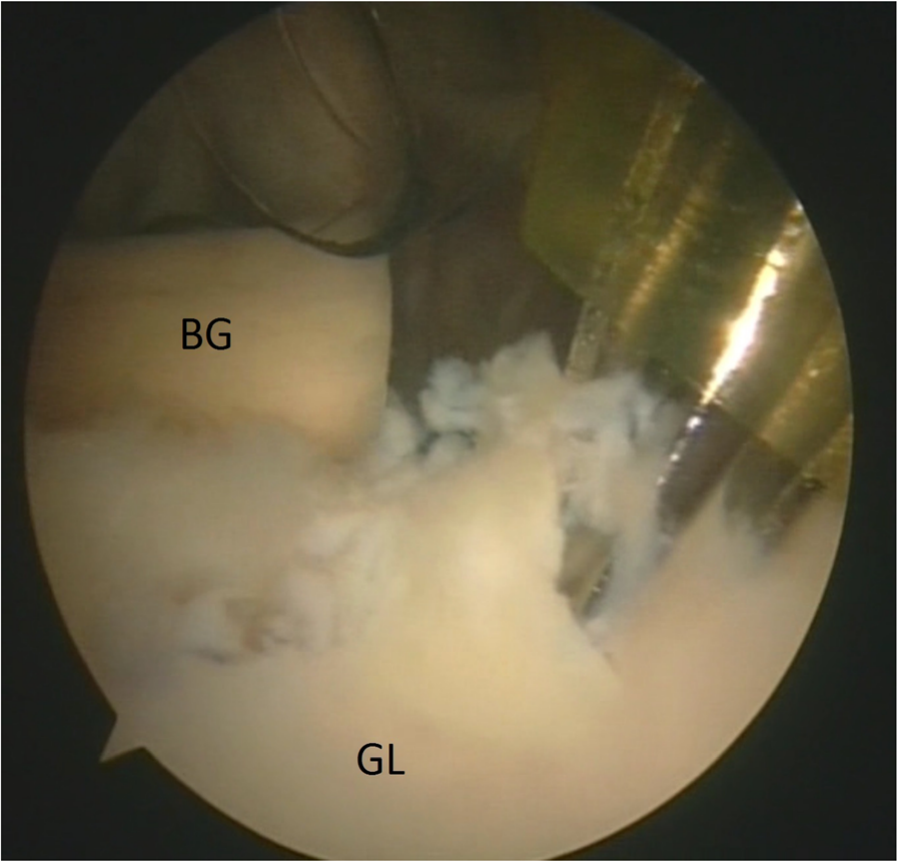
Left shoulder, anterior on the top and posterior on the bottom, scope from posterior portal, drill from antero-superior portal. Drill hole at 10 o’clock position for the anchor (BG: bone graft, GL: glenoid)

**Fig. 7:**
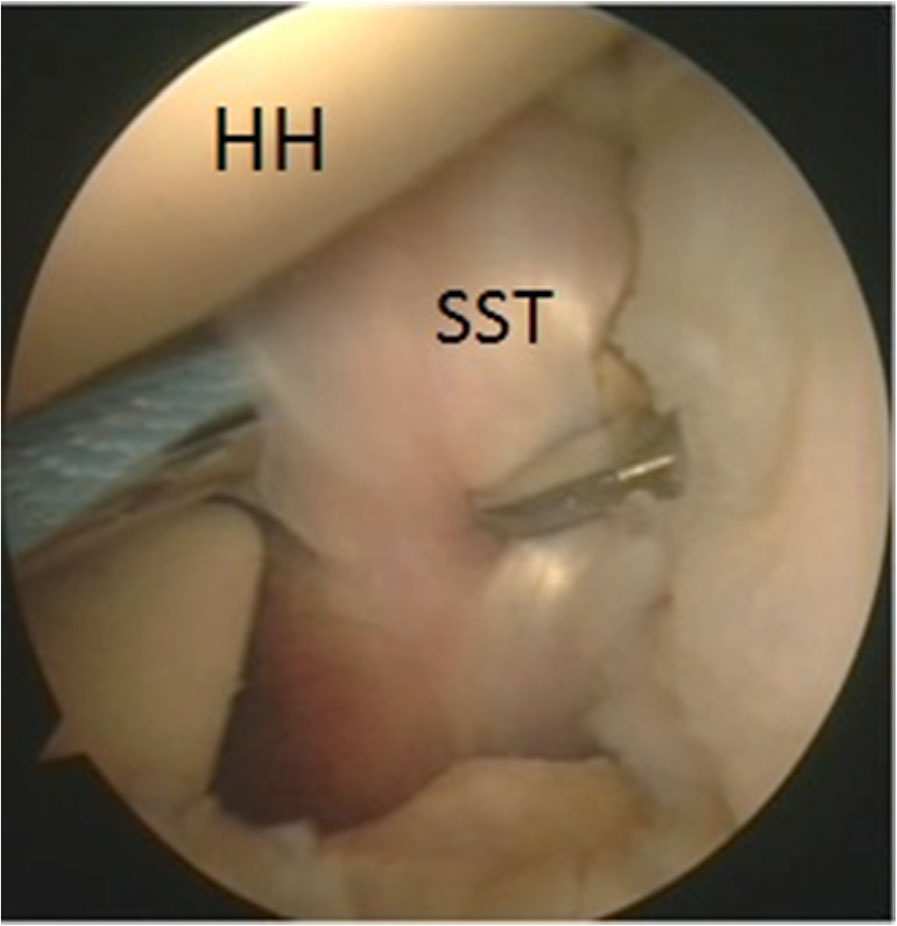
Left shoulder, anterior on the right and posterior on the left, scope from posterior portal, suture passing device from antero-inferior portal. The upper third of the subscapularis tendon is penetrated with a tape (HH: humeral head, SST: subscapularis tendon)

**Fig. 8:**
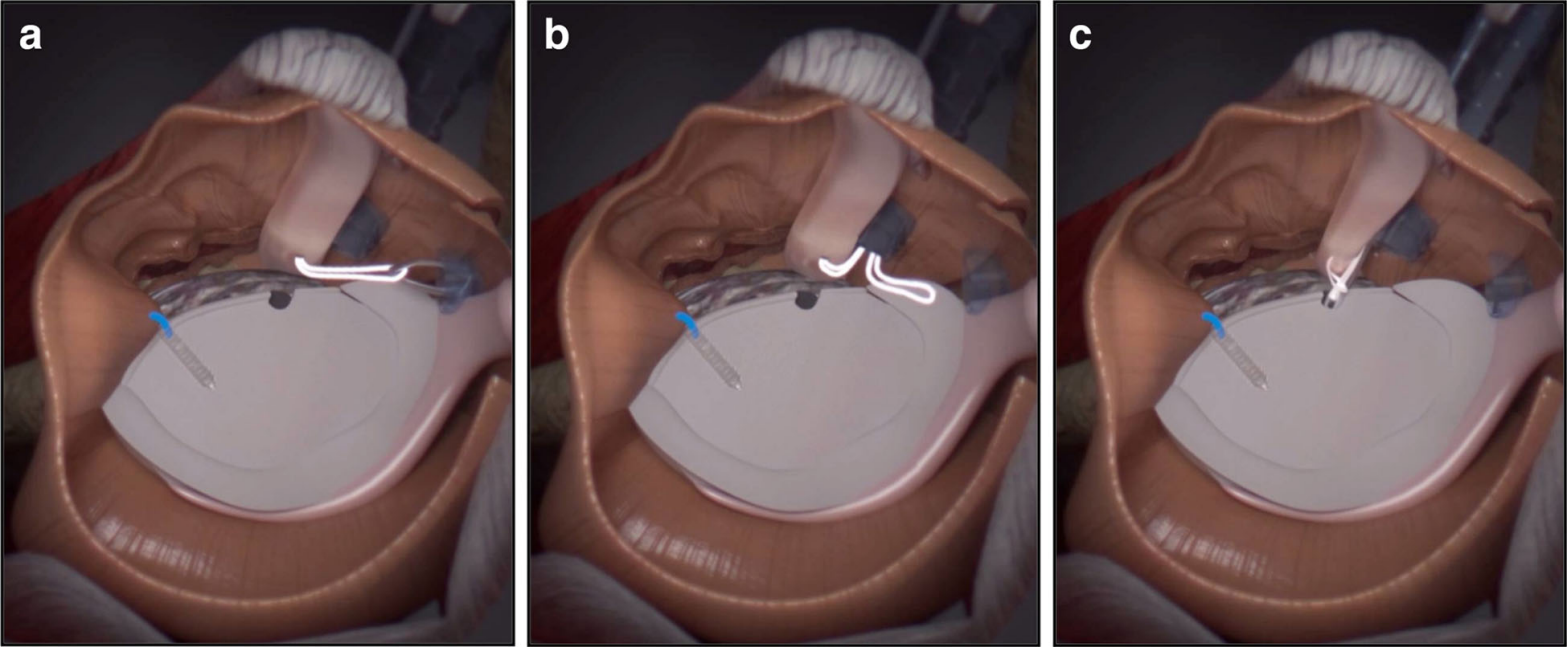
The tape is carried out through the upper cannula with a suture retriever (**a**) and then passed again in the lower cannula (**b**). A loop was created, and both free ends of the tape were passed through the anchor’s eyelet and the anchor was pushed along the tape toward the bone hole (**c**)

**Fig. 9:**
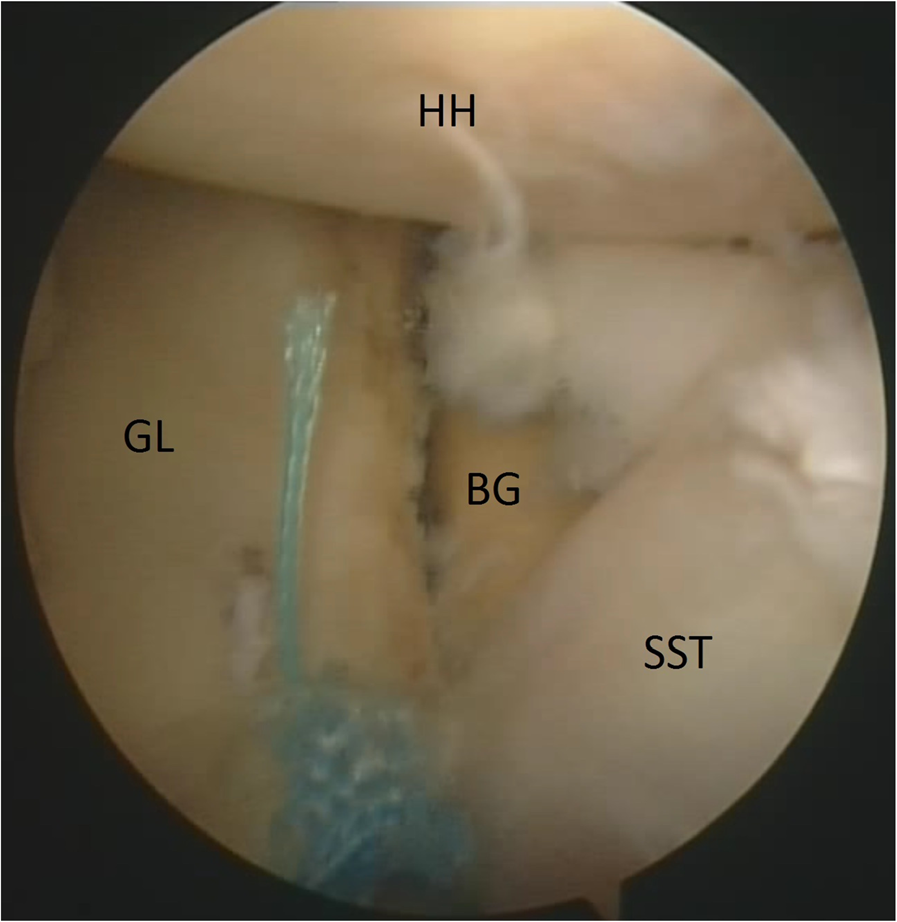
Left shoulder. Final view from the antero-superior portal showing the closure of the anterior pounce on the bone graft and subscapularis tenodesis (HH: humeral head, GL: glenoid, BG: bone graft, SST: subscapularis tendon)

The upper third of the subscapularis tendon - as described in the original technical paper (Maiotti & Massoni, 2013) - is usually fixed at 3 (R) or 9 (L) o’ clock positions on the glenoid neck. In this technique**,** the tenodesis bone holes are placed on the anterior glenoid edge in an upper position (at 10 o’clock in the left shoulders and 2 o’clock in the right shoulders) (Fig. 6) in all cases to avoid possible interference with the upper glenoid tunnel used for the fixation of the graft. A second reason is that by elevating the subscapularis, it is possible to shift the inferior capsulolabral complex up to get a better covering of the graft. The middle upper third of the subscapularis tendon was penetrated approximately 5 mm from its superior border with a suture passing device loaded with tape (Ultra Tape; Smith & Nephew) just over the graft (Fig. 7). Then, one of the free end is passed out through the upper cannula with a suture retriever and then passed again into the lower cannula (Fig. 8a-b). A punch device proved extremely useful to assess the direction and depth of the anchor bone hole. At this point, a loop was created, and both free ends of the tape were passed through the anchor’s eyelet (2.9 mm Bioraptor, Smith & Nephew); then, the anchor was pushed along the tape toward the bone hole (Fig. 8c). While the anchor was inserted into the bone, the tape sutures were kept in traction in a parallel position, and care was taken to keep the specimen’s arm in neutral rotation to avoid excessive tension on the tissue repair. It was important to control the insertion of the anchor’s eyelet and tape, thereby maintaining the correct direction before impacting. Advancement of the subscapularis tendon over the graft, effective closure of the anterior pouch and a posterior shifting of the humeral head in a correct position centered on the glenoid socket could be clearly visible and assessed by arthroscopic examination from the posterior and antero-superior portals (Fig. 9). For a good covering of the graft, capsular residue and ligaments should be able to make it almost extracapsular. After ASA procedure, graft and tenodesis stability were tested using a probe and humeral head stability and position were evaluated.

All seven specimens were dissected at the end of the two procedures to verify the graft stabilization anteriorly and posteriorly with the four buttons using a grasper, to assess the covering of the tendon on the graft and its position on the glenoid edge, and to identify any interference between the two glenoid tunnels and the anchor for the subscapularis tenodesis; the contact surface between the anterior part of the glenoid and the bone graft was measured using a ruler. Finally, we investigated the neurovascular structures in relation to the gleno-humeral joint.

All procedures were performed by 2 accredited and experienced surgeons with specific skills in shoulder arthroscopy and on specimen study (RR and MM).

## Results

The average time required to prepare the graft was 30 min; this time is avoided in the case in which we used the preformed xenograft. The average time to perform the ASA-BB procedure on the specimens was 112 min (80–150 min); the shorter time was obtained using the xenograft. The management of the sutures and buttons through the graft and glenoid tunnel was not difficult. In one case, it was necessary to flush out the graft at the glenoid level using a motorized burr. We have had no complications or fractures in any cases.

**Fig. 10:**
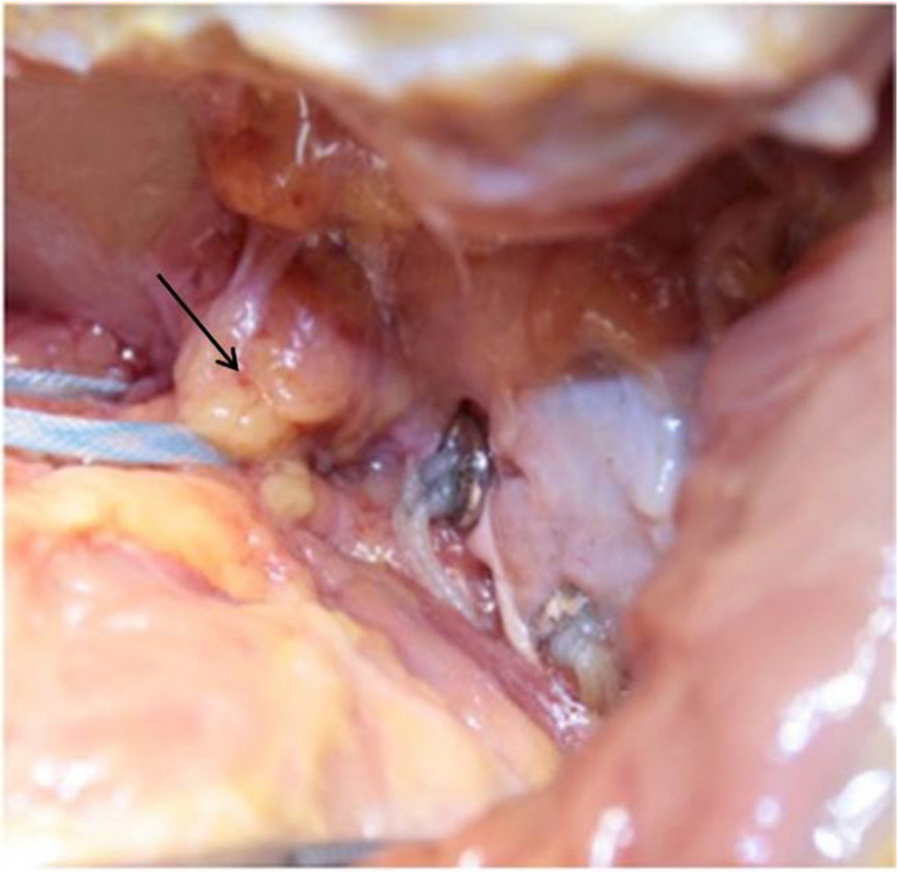
Posterior side of the scapular neck: absence of interference of the upper graft fixation button with suprascapular nerve (arrow)

**Fig. 11:**
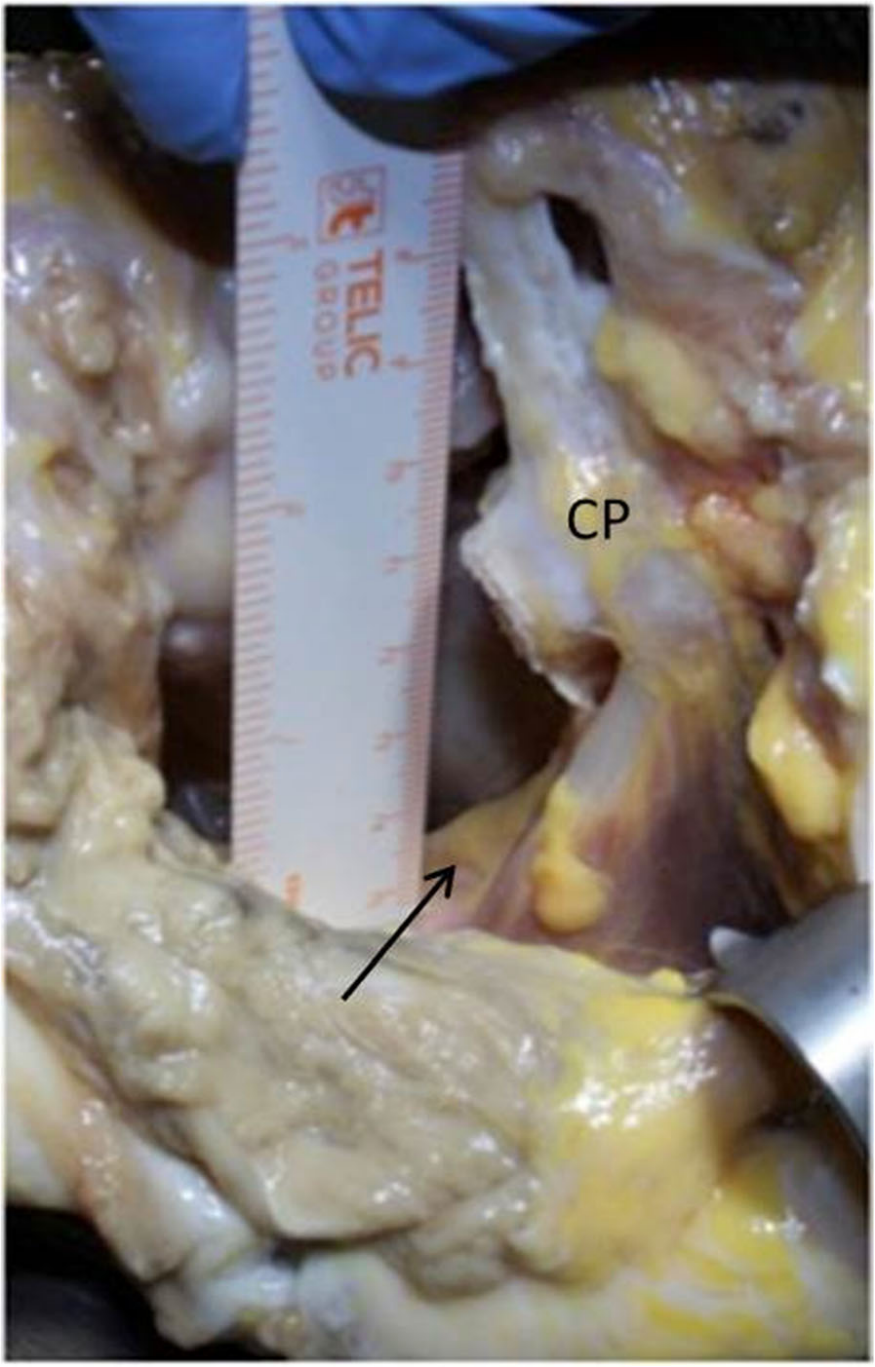
Anterior aspect of muscolo-cutaneus nerve (arrow) showing the distance from the antero-inferior portal enlarged from seven mm to fiftheen (CP: coracoid process)

No interferences were noticed between portals and the cephalic vein anteriorly. The center of the graft was located in a subequatorial position. The contact surface between the anterior part of the glenoid and the bone graft was 80% of the surface area of the bone block in the first 2 cases and 90% in the other 4 cases. No evidence was found of soft tissue interposition between the bone graft and the glenoid. All the grafts were positioned 1 mm more medial with respect to the articular surface. No interference was found between graft tunneling and the anchor side for the subscapularis tenodesis. There was a good covering of the graft by the tendon and a good position of its fixation on the glenoid neck. At the end of bone block procedure the shoulder was not easily dislocable as before the procedure in presence of a glenoid bone defect of about 25%. After ASA procedure the humeral head resulted recentered and shifted posteriorly under arthroscopic view. There was no vascular damage to the anterior and posterior vessels. No interference with the axillary nerve anteriorly and the suprascapular nerve posteriorly was noted. The axillary nerve was located 35 mm to 55 mm (average 40.3 mm) from the inferior gleno-humeral portal. The suprascapular nerve was located 8 mm to 15 mm (average 11 mm) from the two glenoid holes (Fig. 10). The musculocutaneous nerve was located 33 mm to 50 mm (average 38 mm) from the insertion of the coraco-biceps tendon (Fig. 11).

## Discussion

This paper demonstrates the feasibility and reproducibility of the first all arthroscopic procedure to combining a glenoid bone graft augmentation according to Taverna (Taverna et al., 2014) and a partial subscapularis tenodesis according to Maiotti (Maiotti & Massoni, 2013) to treat recurrent complex anterior instability associated with glenoid bone loss of about 25% and capsular inconsistency. The optimal treatment of this pathology, especially if associated with a glenoid bone defect, has not yet been entirely established (Symeonides, 1989; An et al., 2016; Kempf et al., 1999; Morrey & Janes, 1976; Rowe et al., 1978; Wymenga & Morshuis, 1988). The open trans-glenoid Bankart reinsertion was considered for years the “gold standard” for treating recurrent instability and has given excellent results, with a recurrence rate of 1%. However, broad use of this technique, arthroscopically assisted using anchors and popularized as the Bankart repair, remains more controversial because of the relatively high number of recurrences of 20% to 64%, especially if associated with glenoid bone defects (Alkaduhimi et al., 2016; Burkhart & De Beer, 2000; Cole & Warner, 2000; Field et al., 1999; Morrey & Janes, 1976; Bankart, 1938). This clinical survey was supported by the biomechanical and clinical retrospective studies (Arciero et al., 2015; Longo et al., 2014).

In the current and previous decade, the literature has focused on the debate concerning the open and arthroscopic Latarjet procedure, and has supported this intervention to solve three main problems: glenoid bone loss, an engaging Hill-Sachs lesion and capsular insufficiency. The results for this procedure of recurrences at medium- and long-term follow up were between 0 and 5% (Alvi et al., 2016; Degen et al., 2016; Kleiner et al., 2016; Lafosse et al., 2007; An et al., 2016; Cassagnaud et al., 2003; Matton et al., 1992; Russo et al., 1990; Russo et al., 1998; Torg et al., 1987; Di Giacomo et al., 2011; Kany et al., 2016; Ramhamadany & Modi, 2016). This technique can be considered a non-anatomical procedure and is not without risks. Furthermore, results regarding the percentage of coracoid bone graft healing and the incidence of secondary gleno-humeral osteoarthritis seem to be less favorable. In particular, increased use of the arthroscopic Latarjet technique would increase peri-operative and post-operative complications to between 20% and 40% (Griesser et al., 2013). That is why other solutions are being considered to overcome the inherent technical difficulties in transferring the coracoid graft onto the glenoid (Matton et al., 1992; Di Giacomo et al., 2011; Athwal et al., 2016; Gartsman et al., 2017; Guity et al., 2002; Randelli et al., 2016; Young & Rockwood, 1991). A novel, all-arthroscopic technique using three cortical free bone grafts has been described using 4 buttons to stabilize the graft from back to front to avoid complications related to anterior screw fixation and anatomical modification of the coracoacromial arch (Taverna et al., 2008; Taverna et al., 2014). For our study, we chose the percentage of 25% because this is the glenoid defect size for which in literature it is well known that shoulder has to be treated with a bone block procedure because the standard anterior capsulorrhaphy is not indicated (Burkhart & De Beer, 2000; Provencher et al., 2010).

With this new technique, after the stabilization of the anterior glenoid graft, a Bankart repair is mandatory to center the head on the glenoid fossa. In cases of recurrent instability, the percentage of anterior capsule inconsistency can be very high, and this problem can create conditions not conducive to obtaining a good result. In 2013, it was proposed to treat recurrent instability with a moderate bone glenoid defect and capsular deficiency with a Bankart repair associated with an ASA technique (Maiotti & Massoni, 2013). In 2016, the first short term follow up series reported very good clinical results with recurrences of 2.5% without impairment of external rotation (Maiotti et al., 2016). The good biomechanical effect of the upper subscapularis tenodesis (Klungsøyr et al., 2015), as an anterior barrier in recentering the humeral head and a good sliding effect of the tendon with the arm in abduction, was demonstrated in a biomechanical study on specimens (Schröter et al., 2016). In a publication of a multicenter study, the reproducibility of this combination of treatments using the Bankart repair and the ASA technique was confirmed showing good results relative to failures and external rotation (Maiotti et al., 2017). Our aim was to demonstrate that the association of a free bone block using four buttons and the ASA technique to treat complex recurrent instability with glenoid bone loss and capsular insufficiency could be feasible and reproducible. We focused on glenoid defect in order to have less variables, without considering humeral head defects also because it is difficult to verify the position and the shape of the Hill Sachs lesion arthroscopically. We observed arthroscopically at the end of the procedure that the humeral head was recentered, even if an inferior gleno-humeral ligament reconstruction was not done. The association of both procedures can be easily performed after an adequate training by surgeons having a good skills level for arthroscopic shoulder surgery. No anatomical interference was observed between the two tunnels for the bone block stabilization and the subscapularis tenodesis hole anchor. In our opinion, the stabilization of the bone graft with four buttons can certainly be considered an improvement over the use of screws, and the procedure avoids the risk of mechanical contact with the humeral head and a non-orthogonal pressure on the fixation strength of the graft on the glenoid bone defect while in this procedure graft compression is perpendicular and not oblique allowing a better healing. We could verify in all specimens the high stability of the graft on the glenoid without any micro-motion. In all procedures, the partial subscapularis tenodesis allows the recentering of the head, pushing it posteriorly and acting in a way opposite to that of the Remplissage technique, creating a lift up effect on the inferior capsule. The procedure achieved closure of the axillary pouch and increased the contact between the graft, the subscapularis muscle and the residual capsule. We noticed no complications to the main vessels and nerves around the joint anteriorly and posteriorly using the four portals and the dedicated new instrumentation. In particular, the distance between the mid-glenoid portals of the 15 mm cannula can be considered not a risk for axillary nerve injury because it is 40 mm superior to the nerve. On the back side, the superior button for graft stabilization is less than 1 cm from the suprascapular nerve and can thus be used with relative safety, even though it might be at a relative risk. In conclusion, this new proposal of an all arthroscopic bone grafting procedure with concomitant soft tissue reconstruction using the upper part of the subscapularis tendon on specimens showed good stabilization of the shoulder in patients with combined glenoid defect of about 25% and capsular insufficiency. This procedure can be considered safe with respect to the risk of nerve damage, in the same manner as a simple Bankart repair anteriorly and posteriorly. Furthermore, we think that this new technique could be a feasible and reproducible alternative to the arthroscopic or open Latarjet procedures for patients with bone defect associated to capsular insufficiency practicing contact sports. In fact the effect of tenodesis could be comparable to that of the conjoint tendon in Latarjet procedure. Moreover this procedure is a low-risk technique, that can be performed using only three portals and not five as for the arthroscopic Latarjet; graft compression is perpendicular and not oblique allowing a better healing; the present technique is more anatomic, not modifying the coraco-acromial arch. Finally, the use of a preformed graft consistently reduced the surgical time.

Given that this combined arthroscopic technique (bone block associated with ASA) is safe and easily reproducible, it is our intent to conduct a clinical study. It is essential that this technique should be evaluated clinically before its application on a large scale.

### Limitations

This study has several limitations. The number of specimens treated is very small, and both techniques were applied to the shoulders using specimens non-homogeneous by age, sex and non-comparable glenoid bone defects; no study for measuring the glenoid version of the specimen was done. New biomechanically detailed studies should be carried out, examining, for example, modification of external rotation, the friction between the subscapularis tendon and the graft, the humeral head position relative to the glenoid rotational center after the removal of the capsulolabral complex and after the creation of a GBL of about 25%. This would allow the evaluation of the recentering effect of the subscapularis tenodesis alone or in association with the bone block in different arm positions, from 0° to 60° of elevation. Moreover, all anatomical structures should be subjected to cycles of movement to test the biomechanics of the head in this new anatomical position, considering also the strength of the fixation systems for both bone and tendon. Last but not least, knowledge is very limited on the healing of the free bone block using the button system and on the healing of the subscapularis tendon on the glenoid rim.

## Conclusion

This is the first cadaver study to test the feasibility and safety of this new all-arthroscopic combined technique with bone block and ASA for treating gleno-humeral instability with glenoid bone loss of about 25% and anterior capsule-labral deficiency. Further studies have to be performed to assess the stable fixation of the bone graft, the re-centring of the humeral head and the restoration of shoulder stability and function.
